# Association of the rs2814778 variant in the *ACKR1* gene, responsible for the Duffy erythrocyte antigen "null" phenotype, with COVID-19 severity in Southern Brazil

**DOI:** 10.31744/einstein_journal/2026AO1543

**Published:** 2025-12-05

**Authors:** Kelly Silvério Góis, Matheus Braga, Victor Hugo de Souza, Julyane Schavaren, Sergio Grava, Andréa Name Colado Simão, Jeane Eliete Laguila Visentainer, Quirino Alves de Lima

**Affiliations:** 1 Universidade Estadual de Maringá Post-Graduate Program in Biosciences and Pathophysiology Department of Clinical Analysis and Biomedicine Maringá PR Brazil Post-Graduate Program in Biosciences and Pathophysiology, Department of Clinical Analysis and Biomedicine, Universidade Estadual de Maringá, Maringá, PR, Brazil.; 2 Universidade Estadual de Londrina Research Laboratory in Applied Immunology Department of Pathology Londrina PR Brazil Research Laboratory in Applied Immunology, Department of Pathology, Clinical Analysis and Toxicology, Universidade Estadual de Londrina, Londrina, PR, Brazil.; 3 Universidade Estadual de Maringá Immunogenetics Laboratory Department of Basic Sciences of Health Maringá PR Brazil Immunogenetics Laboratory, Department of Basic Sciences of Health, Universidade Estadual de Maringá, Maringá, PR, Brazil.

**Keywords:** Coronavirus, SARS-CoV-2, COVID-19, Duffy blood-group system, ACKR1 protein, human, Polymorphism, single nucleotide

## Abstract

**Objective:**

This study aimed to analyze the possible association between rs2814778, rs12075 ( *ACKR1* gene), and rs4073 ( *CXCL8* gene) single nucleotide variants and COVID-19 severity.

**Methods:**

This cross-sectional study included 319 COVID-19 diagnosed patients at two hospitals in Paraná, Brazil between 2020 and 2021. Among them, 171 cases were classified as severe or critical and 148 were classified as non-severe. Genotyping was performed using polymerase chain reaction.

**Results:**

We found an association between the rs2814778 variant of the *ACKR1* gene and COVID-19 severity. The C allele in both the T/C and C/C genotypes was identified as a risk factor for severe COVID-19, independent of sex, age, smoking status, cardiovascular disease, diabetes, or obesity. No evidence of an association was observed for the other variants.

**Conclusion:**

The presence of the C allele in the rs2814778 variant indicated an increased risk of severe or critical COVID-19 in the southern Brazilian population across all possible genotypes and genetic inheritance models.

## Highlights

The rs2814778 C allele is linked to severe/critical COVID-19.

The association is independent of major clinical risk factors.

The Duffy "null" phenotype may promote lung inflammation.

rs12075 and rs4073 showed no association with COVID-19 severity.

## Summary

The rs2814778 (-67T>C) variant in the ACKR1 gene, responsible for the Duffy "null" phenotype, was associated with a higher risk of severe/critical COVID-19 in Southern Brazil, regardless of age, sex, smoking status, cardiovascular disease, diabetes, or obesity. The rs12075 and rs4073 variants showed no association.

## Graphical abstract

**Figure:**
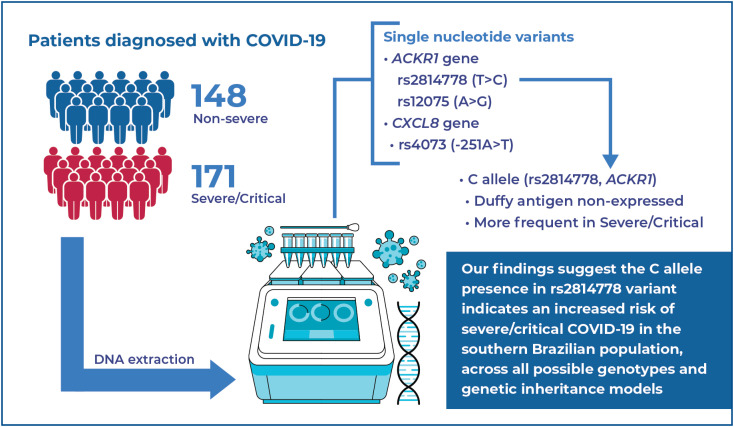

## INTRODUCTION

COVID-19 is a disease caused by the novel coronavirus (SARS-CoV-2), which emerged in China in December 2019 and rapidly spread worldwide.^( 1 , 2 )^ It has become a pandemic that threatens public health and has led to the immediate mobilization of the scientific community, bringing new advances in understanding the role of the immune response in COVID-19. One of the most striking features of SARS-COV-2 infection is its wide range of clinical manifestations, from asymptomatic cases to mild clinical symptoms, or progression to severe clinical conditions and death.^( 3 )^

Most patients with severe COVID-19 report pulmonary and systemic inflammation associated with high cytokine and chemokine levels.^( 4 , 5 )^ Excessive systemic inflammatory responses can lead to the development of Acute Respiratory Distress Syndrome (ARDS) and the dysfunction or failure of multiple organs and systems.^( 6 )^ High levels of chemokines, such as interleukin-8 (IL-8), contribute to excessive neutrophil recruitment from the circulation into the lungs, which are overwhelmed by the severe systemic inflammation of COVID-19.^( 7 , 8 )^

IL-8 is a CXC chemokine that plays a crucial role in the immune response by stimulating neutrophil migration.^( 9 )^ This chemokine is encoded by the *CXCL8* gene, and among its various single-nucleotide variants (SNVs), the most studied is rs4073 (-251A>T), located in its upstream region, where adenine (A) is replaced by thymine (T).^( 10 )^ This SNV has been associated with increased susceptibility to inflammation and severe infections.^( 11 )^ The Duffy antigen receptor for chemokines (DARC) is an important erythrocyte antigen that selectively binds chemokines, especially IL-8, with high affinity, but does not signal like other seven-transmembrane receptors. It acts as a chemokine reservoir, regulating inflammation and altering the concentration of soluble chemokines in blood and tissue compartments.^( 12 )^

The Duffy antigen is expressed not only in erythrocytes but also in venular and postcapillary endothelial cells, lungs, esophagus, and adipose tissue.^( 13 )^ The main Duffy antigens, A and B, are encoded by the FY*A (G) and FY*B (A) alleles of the *atypical chemokine* *receptor 1* ( *ACKR1* ) gene, which is characterized by the polymorphism rs12075 (c.125A>G). Interestingly, the rs2814778 (-67T>C) SNP located in the 5’ untranslated region (UTR) of the FY*B allele in the *ACKR1* gene, suppresses erythrocyte expression of the Duffy antigen. This phenomenon occurs in a significant proportion of individuals of African descent and results in the "Duffy null" phenotype.^( 13 )^

## OBJECTIVE

This study aimed to analyze the possible association between three SNVs and COVID-19 severity: rs2814778 (-67T>C), a 5’ untranslated region variant in the *ACKR1* gene; rs12075 (c.125A>G), an exonic variant in the *ACKR1* gene; and rs4073 (-251A>T), an upstream variant in the *CXCL8* gene.

## METHODS

### Study population

This cross-sectional study included 319 patients diagnosed with COVID-19 between 2020 and 2021 using real-time Reverse Transcription Polymerase Chain Reaction (RT-qPCR). Participants were recruited from two hospitals in Paraná, Brazil: *Hospital Universitário de Londrina* and *Hospital do Paraná* , Maringá. Of these participants, 171 were classified as having severe or critical illness, characterized by room air oxygen saturation below 90%, clinical signs of pneumonia, severe respiratory distress, ARDS, or other conditions requiring life support, such as invasive or non-invasive mechanical ventilation. The remaining 148 patients were categorized as having non-severe COVID-19, as defined by the absence of severe or critical criteria according to World Health Organization (WHO) guidelines.^( 14 )^

This study was approved by the Standing Ethics Committee of the *Universidade Estadual de Maringá* (CAAE: 38095420.5.0000.0104; # 4.357.896) and Standing Ethics Committee of the *Universidade Estadual de Londrina* (CAAE: 31656420.0.0000.5231; #4.053.033). All participants provided written informed consent (TCLE).

### Sample collection and DNA extraction

Peripheral blood samples were collected from patients using EDTA anticoagulant tubes. Genomic DNA was then obtained by Biopur Kit® (BIOMETRIX Diagnostic, Curitiba, PR, Brazil), following the manufacturer's instructions. DNA concentration and quality were assessed using NanoDrop 2000® equipment (Wilmington, USA).

### Genotyping

#### rs2814778 of the *ACKR1* gene

The rs2814778(-67T>C) variant was genotyped using polymerase chain reaction (PCR), followed by fragment analysis with the *StyI* enzyme through PCR-restriction fragment length polymorphism (PCR-RFLP), according to a previously described protocol.^( 15 )^ The primers sequences are listed in Table 1S (Supplementary Material) .

The amplified fragments were verified by electrophoresis on a 2% agarose gel at 150 V for 10 min using SYBR Safe DNA gel stain (Invitrogen). The digested fragments were analyzed on a 3.5% agarose gel at 70 V for 45 min and then at 80 V for 20 min. Both fragments were documented using a photo documentation software.

#### rs12075 of the *ACKR1* gene

The FY*A and FY*B alleles (G>A) were genotyped using the polymerase chain reaction sequence-specific primer (PCR-SSP) technique, following a previously described protocol.^( 16 )^ The primers sequences are listed in Table 2S (Supplementary Material) . After amplification, the final PCR products were visualized by electrophoresis on a 2% agarose gel at 150 V for 20 min using SYBR Safe DNA gel stain (Invitrogen). The fragments were recorded using photodocumentation.

#### rs4073 of the *IL8* gene

The (-251A>T) variant of the *CXCL8* gene was determined by real-time polymerase chain reaction (qPCR) using the pre-designed TaqMan SNP Genotyping Assay C__11748116_10, Single Nucleotide Polymorphism (SNP) (Applied Biosystems, Waltham, MA, USA). This assay contains primers and fluorescent-labeled probes (VIC and FAM) with a minor groove binder (MGB) for allele detection. TaqPath™ ProAmp™ Master Mix (Applied Biosystems, Waltham, MA, USA) was used for qPCR following the manufacturer's protocol. The reaction conditions were as follows: pre-read at 60°C for 30 s, initial denaturation at 95°C for 10 min, denaturation at 92°C for 15s for 50 cycles, primer annealing and extension at 60°C for 1 min for 50 cycles, and a final extension at 60°C for 30s. Amplification products were visualized and analyzed using StepOne™ Software v2.3.

### Statistical analysis

The sample size was calculated using the QUANTO 1.2.4 software ( http://biostats.usc.edu/software ). Statistical analysis of patients’ characteristics was performed using R 4.2.1 software, applying χ²-tests and *t* -tests. Genetic statistical analyses and Hardy-Weinberg equilibrium (HWE) testing were conducted using SNPStats software ( https://www.snpstats.net/start.htm )^( 17 )^ SNPStats was also used to determine the odds ratio (OR) with 95% confidence intervals (CIs) using χ²-tests and logistic regression. Differences were considered statistically significant at p<0.05.

## RESULTS

Significant differences were observed between the non-severe and severe or critical cases in terms of sex, age, smoking status, and comorbidities, such as cardiovascular disease, diabetes, and obesity ( Table 1 ). These variables were adjusted as potential confounders in the genetic analysis.

**Table 1:** Demographic data of non-severe and severe/critical patients

Characteristics		Non-severe n=148	Severe/Critical n=171	p value
Age		46.68±14.38	67.20±16.14	<0.01 *
Sex				
	Female	94 (0.64)	70 (0.41)	<0.01^‡^
	Male	54 (0.36)	101 (0.59)	
Smoking				
	No	140 (0.95)	134 (0.78)	<0.01^‡^
	Yes	8 (0.05)	37 (0.22)	
CVD				
	No	122 (0.82)	70 (0.41)	<0.01^‡^
	Yes	26 (0.18)	101 (0.59)	
Diabetes				
	No	136 (0.92)	103 (0.60)	<0.01^‡^
	Yes	12 (0.08)	68 (0.40)	
Obesity				
	No	78 (0.53)	128 (0.75)	<0.01 ‡
	Yes	70 (0.47)	43 (0.25)	
Intensive care				
	No	-	67 (0.39)	-
	Yes	-	104 (0.61)	
Intubation				
	No	-	69 (0.40)	-
	Yes	-	102 (0.60)	
ARDS				
	No	-	69 (0.40)	-
	Yes	-	102 (0.60)	
Death				
	No	-	78 (0.46)	-
	Yes	-	93 (0.54)	

* *t* -test;

‡ χ^2^-test.

CVD: cardiovascular disease; ARDS: Acute Respiratory Distress Syndrome.

The genotype frequency distribution of non-severe patients was in HWE (p>0.05), while *ACKR1* gene variants (rs2814778 and rs12075) of severe/critical patients deviated from HWE.

The genotype and allele frequencies of the non-severe and severe/critical cases are shown in table 2 . An association was observed between the rs2814778 variant of *ACKR1* and COVID-19 severity. The C allele, as well as the T/C and C/C genotypes, were identified as risk factors for developing severe COVID-19, independent of sex, age, smoking status, cardiovascular disease, diabetes, and obesity. No associations were found for the other polymorphisms.

**Table 2:** Association between *ACKR1* variants and COVID-19 severity, adjusted for sex, age, smoking status, cardiovascular disease, diabetes, and obesity

Polymorphism		Non-severe n=148	Severe/Critical n=171	OR (95% CI)	p value
rs2814778					
Codominant					
	T/T	138 (0.93)	130 (0.76)	1.00	<0.01 *
	T/C	9 (0.06)	30 (0.18)	4.56 (1.59-13.10)	
	C/C	1 (0.01)	11 (0.06)	21.99 (1.62-297.99)	
Dominant					
	T/T	138 (0.93)	130 (0.76)	1.00	<0.01 *
	T/C-C/C	10 (0.07)	41 (0.24)	5.88 (2.19-15.81)	
Recessive					
	T/T-T/C	147 (0.99)	160 (0.94)	1.00	<0.01 *
	C/C	1 (0.01)	11 (0.06)	17.67 (1.29- 242.42)	
Allele					
	T	285 (0.96)	290 (0.85)	1.00	<0.01 *
	C	11 (0.04)	52 (0.15)	4.61 (1.98-10.74)	
rs12075					
Codominant					
	A/A	54 (0.37)	80 (0.47)	1.00	0.14 *
	A/G	80 (0.54)	63 (0.37)	0.56 (0.30-1.06)	
	G/G	14 (0.09)	28 (0.16)	1.10 (0.43-2.79)	
Dominant					
	A/A	54 (0.37)	80 (0.47)	1.00	0.17 *
	G/A-G/G	94 (0.63)	91 (0.53)	0.66 (0.36-1.19)	
Recessive					
	A/A-G/A	134 (0.91)	143 (0.84)	1.00	0.38 *
	G/G	14 (0.09)	28 (0.16)	1.48 (0.62-3.54)	
Allele					
	A	188 (0.64)	223 (0.65)	1.00	0.58 *
	G	108 (0.36)	119 (0.35)	0.89 (0.58-1.36)	
rs4073					
Codominant					
	T/T	54 (0.36)	51 (0.30)	1.00	0.53 *
	A/T	69 (0.46)	77 (0.45)	1.22 (0.62-2.39)	
	A/A	25 (0.17)	43 (0.25)	1.60 (0.71-3.63)	
Dominant					
	T/T	54 (0.36)	51 (0.30)	1.00	0.36 *
	A/T-A/A	94 (0.64)	120 (0.70)	1.33 (0.72-2.48)	
Recessive					
	T/T-A/T	123 (0.83)	128 (0.75)	1.00	0.36 *
	A/A	25 (0.17)	43 (0.25)	1.43 (0.69-2.97)	
Allele					
	T	177 (0.60)	179 (0.52)	1.00	0.26 *
	A	119 (0.40)	163 (0.48)	1.26 (0.84-1.89)	

* Statistical analysis performed by SNPStats software applying χ²-tests and logistic regression.

OR: odds ratio; 95%CI: 95%confidence interval.

## DISCUSSION

Our study identified an association between the (-67T>C) variant of the *ACKR1* gene and COVID-19 severity. The presence of the C allele indicates a high risk of severe or critical COVID-19 across all possible genotypes and genetic inheritance models. Initial observations from another Brazilian study reported an association between the C allele and COVID-19 severity, in which the Duffy null allele, hypertension, and age were independently associated with the need for hospitalization.^( 18 )^ The (-67T>C) variant in the 5’UTR region of *ACKR1* , where a T>C nucleotide substitution occurs, impairs promoter activity in erythrocytes by disrupting the binding of erythrocyte transcription factor GATA1. This disruption results in a lack of Duffy antigen expression in the erythrocyte membrane.^( 19 )^

Normally, the Duffy antigen binds to multiple inflammatory chemokines, acting as a chemokine reservoir that helps regulate plasma levels and potentially dampens leukocyte activation.^( 20 , 21 )^ In the absence of the Duffy erythrocyte antigen (Duffy-null phenotype), a pro-inflammatory effect may occur by promoting leukocyte migration to the lungs. Without Duffy erythrocyte antigens, inflammatory chemokines become available to bind to endothelial Duffy, leading to inflammatory cell migration, and consequently, increased endothelial and epithelial damage.^( 22 )^

A study involving African American participants found that patients with acute lung injury (ALI) who were Duffy deficient had a 17% higher risk of death and a greater need for mechanical ventilation than those who expressed the Duffy antigen.^( 23 )^ Furthermore, neutrophil migration into the alveolar space increased in Duffy erythrocyte-knockout animals, along with elevated levels of CXC chemokines, when acute respiratory failure was induced by inhaled lipopolysaccharide.^( 24 )^ As suggested by Hebbel and Vercellotti, the absence of Duffy red blood cell antigens may be a risk factor for severe COVID-19.^( 25 )^

Historically, DARC in erythrocytes has been considered a minor blood group and a receptor for *Plasmodium vivax* , the causative agent of malaria.^( 26 )^ The C/C genotype, known as the "Duffy null" state, is thought to confer a survival advantage, leading to positive selection for this phenotype in West African populations, suggesting a strong role for natural selection.^( 27 )^ This could explain the deviation in HWE in severe/critical cases, as natural selection can bias the genotype frequency of HWE expectations.^( 28 )^ The (-67T>C) variant of *ACKR1* is haplotypically associated with the FY*B allele of the rs12075 (c.125A>G) polymorphism in the same gene.^( 29 )^ At the population level, linkage disequilibrium (LD) refers to the residual association between SNV-specific alleles on a chromosome that has not been disrupted by historical recombination events.^( 30 )^

Our study found no association between COVID-19 severity and the Duffy antigens FY*A (G) and FY*B (A) of rs12075 (c.125A>G) in *ACKR1* . To our knowledge, this is the first study to evaluate this polymorphism in the COVID-19 context. This variant is located in the exon of *ACKR1* , where a nucleotide replacement change (G>A) occurs, resulting in a single amino acid change, replacing a glycine residue with an aspartic acid residue (Gly42Asp).^( 21 )^ In a genome-wide association analysis, rs12075 (Asp42Gly) was identified as a major regulator of DARC-mediated cytokine binding in erythrocytes, affecting the circulating concentrations of several proinflammatory cytokines. This variant accounted for approximately 20% of the variability in serum monocyte chemoattractant protein-1 (MCP-1) concentrations and was associated with serum levels of interleukin-8 and RANTES, suggesting a functional change responsible for cytokine binding.^( 31 )^ Another study reported an association between the Fya blood type and low serum levels of CXCL6, CXCL5, CCL11, CXCL1, CCL2, CCL13, and CCL17.^( 32 )^

Some studies have linked the rs12075 variant to inflammatory diseases. The FY*B allele (AG/GG genotype) has been associated with protection against severe *Plasmodium falciparum* malaria and hospitalization.^( 33 )^ In breast cancer, the GG genotype is correlated with a higher risk of recurrence, particularly in triple-negative cases.^( 34 )^ In chronic hepatitis C, AG/GG genotypes have been associated with slower liver fibrosis progression and reduced cirrhosis risk.^( 35 )^ Ho­wever, they do not appear to be directly associated with hepatitis C infection severity^( 36 )^ or the clinical outcome of HTLV-1 infection.^( 37 )^ Similarly, we found no significant association with COVID-19, suggesting that this variant is not associated with viral infections.

The rs4073 (-251A>T) upstream variant of *CXCL8* is located in the promoter region of the gene in an area with histone marks linked to transcriptional activation, such as H3K4me1, which potentially affects gene transcription levels.^( 38 )^ The A allele is associated with higher systemic IL-8 levels in patients with ARDS.^( 39 )^ Additionally, the AA genotype (-251A>T) has been linked to increased *CXCL6* expression (eQTL — expression quantitative trait loci) in visceral adipose tissue, pancreas, and cultured fibroblast cells.^( 40 )^

Our study found no association between the rs4073 (-251A>T) variant and COVID-19 severity in our population, making it the first study to evaluate this variant in relation to disease severity. A Saudi study reported an association between the A allele and an increased risk of COVID-19 susceptibility compared with patients with potentially exposed controls. Although the same study linked the A allele to mortality risk, it did not provide detailed results, polymorphism frequencies between survivors and deaths, or the corresponding P values.^( 41 )^

Our study has some limitations. Although we identified an association between the rs2814778 (-67T>C) variant of *ACKR1* and COVID-19 severity, we were unable to measure IL-8 serum levels in the analyzed patients. Thus, we could not conclusively determine whether this polymorphism directly influenced cytokine levels or neutrophil inflow into the lungs. Additionally, these variants should be investigated in other populations, because their frequencies may vary across different ethnic groups.

Although our study found no significant association between the rs12075 (c.125A>G) and rs4073 (-251A>T) variants and COVID-19 severity, this finding may reflect a true lack of effect in our population. However, we cannot fully exclude the possibility that limited statistical power, allele frequency distribution, or admixed genetic background of the Brazilian population may have reduced the ability to detect subtle associations. These variants may also have population-specific effects that differ in magnitude or direction across ancestries, highlighting the importance of replication in larger and more diverse cohorts.

The deviation from HWE observed in severe/critical cases may reflect the effect of natural selection on the Duffy null allele. Alternatively, this may have resulted from population stratification or sample composition given the admixed nature of our cohort. Our study population primarily consisted of individuals of European descent (80.6%) with smaller contributions from African (12.5%) and indigenous (7.0%) ancestries.^( 42 )^ Nevertheless, we observed a strong association between the rs2814778 (-67T>C) variant of *ACKR1* and COVID-19 severity, suggesting its potential as a promising genetic marker. However, further experimental studies are required to validate these findings.

## CONCLUSION

Our findings indicate that the C allele, as well as the T/C and C/C genotypes of the rs2814778 (-67T>C) variant in *ACKR1* , are risk factors for developing severe COVID-19, independent of sex, age, smoking status, cardiovascular disease, diabetes, and obesity. The rs12075 (c.125A>G) variant in *ACKR1* and rs4073 (-251A>T) in *CXCL8* were not associated with COVID-19 severity.

## supplementary-material

SUPPLEMENTARY MATERIAL

## DATA AVAILABILITY

Data are available to reviewers upon request.
