# Supplementary material for: Association of the rs2814778 variant in the ACKR1 gene, responsible for the Duffy erythrocyte antigen "null" phenotype, with COVID-19 severity in Southern Brazil
Source: Einstein (Sao Paulo). 2025 Dec 5;24:eAO1543. doi: 10.31744/einstein_journal/2026AO1543 (PMC12714069; doi:10.31744/einstein_journal/2026AO1543)
Supplement: SUPPLEMENTARY MATERIAL [file 2317-6385-eins-24-eAO1543-suppl1.pdf]

SUPPLEMENTARY MATERIAL

Association of the rs2814778 variant in the *ACKR1* gene, responsible for the Duffy erythrocyte antigen "null" phenotype, with COVID-19 severity in Southern Brazil

Kelly Silvério Góis, Matheus Braga, Victor Hugo de Souza, Julyane Schavaren, Sergio Grava, Andréa Name Colado Simão, Jeane Eliete Laguila Visentainer, Quirino Alves de Lima Neto

DOI: 10.31744/einstein_journal/2026AO1543

Table 1S. Sequence of *primers* for PCR-RFLP of rs2814778

Primers	Sequence	bp
FYN1	CAAGGCCAGTGACCCCCATA	T: 81<br>C: 61
FYN2	CATGGCACCGTTTGGTTCAG	

bp: base pairs of the digestion product.

Table 2S. Sequence of *primers* for PCR-SSP rs12075

Primers	Sequence	bp
Duffy		180 bp
*FY*A allele*	CAGCTGCTTCCAGGTTGCCAC	
*FY*B allele*	CAGCTGCTTCCAGGTTGCCAT	
Common	CAGGAGACTCTTCCGGTGTAAC	
HGH		434 bp
HGH F	TGCCTTCCCAACCATTCCCTTA	
HGH R	CCACTCACGGATTTCTGTTGTGTTTC	

bp: base pairs.
